# Cytomegalovirus Infection in an Adult Patient With Neuromyelitis Optica and Acute Hemorrhagic Rectal Ulcer: Case Report and Literature Review

**DOI:** 10.3389/fimmu.2020.01634

**Published:** 2020-08-04

**Authors:** Jinmei Luo, Xiaowei Shi, Ying Lin, Na Cheng, Yunfeng Shi, Yanhong Wang, Ben-Quan Wu

**Affiliations:** ^1^Medical Intensive Care Unit and Division of Respiratory Diseases, Department of Internal Medicine, Third Affiliated Hospital of Sun Yat-sen University, Guangzhou, China; ^2^Department of Gastroenterology, The Third Affiliated Hospital of Sun Yat-sen University, Guangzhou, China; ^3^Department of Pathology, The Third Affiliated Hospital of Sun Yat-sen University, Guangzhou, China

**Keywords:** case report, cytomegalovirus, immunocompetent, neuromyelitis optica, acute hemorrhagic rectal ulcer

## Abstract

**Background:** Previous infectious or inflammatory events may be involved in the pathogenesis of neuromyelitis optica (NMO), potentially by triggering an autoimmune response. Cytomegalovirus (CMV)-related NMO (CMV-NMO) is rarely reported. Acute hemorrhagic rectal ulcer (AHRU) is a rare disease with a largely unknown pathogenesis. Herein, we reported a co-NMO and AHRU case associated with CMV infection. In addition, we review previously reported cases of CMV-NMO and CMV-AHRU.

**Case presentation:** A 40-year-old female diagnosed with aquaporin4 (AQP4)-IgG^+^ NMO and a poor response to high-dose intravenous methylprednisolone and immunoglobulin, followed by three rounds of plasma exchange was transferred to Third Affiliated Hospital of Sun Yat-sen University, Guangzhou, China. She developed repeated acute lower gastrointestinal hemorrhage from the third day of admission. Abdominal computed tomography angiography (CTA) and interventional angiography did not detect any bleeding vessel. Bedside colonoscopy revealed a large ulcer-like lesion at 10 cm above the anus. Rectal biopsy pathology confirmed a CMV infection on day 23 post-admission, and cerebrospinal fluid (CSF) pathogen gene sequencing detected CMV gene copies on day 25 post-admission. After 2 weeks of treatment with ganciclovir and sodium phosphinate, the patient's lower gastrointestinal bleeding stopped, and her limb muscle strength and visual acuity gradually improved. After 4 weeks of antiviral therapy, colonoscopy showed that the intestinal wall of the original lesion was smooth. Hematoxylin and eosin (HE) staining and immunohistochemistry (IHC) of a biopsy specimen was negative for CMV, her right eye vision was normal, and limb muscle strength had recovered. Serum AQP4-IgG was negative, and lesions on brain magnetic resonance imaging (MRI) manifested shrinkage.

**Conclusions:** The benefits of antiviral therapy remain unclear; however, clinicians should be aware of the possibility of CMV-related NMO, if NMO was refractory to high-dose intravenous methylprednisolone, immunoglobulin, and plasma exchange. Moreover, clinicians should consider the possibility of CMV-related AHRU when recurrent acute lower gastrointestinal bleeding occurs in a patient.

## Introduction

NMO is a relapsing, autoimmune inflammatory disorder that typically affects the optic nerves and spinal cord. Approximately two-thirds of cases are associated with AQP4-IgG (1). Treatment for NMO includes high-dose intravenous methylprednisolone and plasma exchanges if corticosteroids are not sufficient. However, a previous study has indicated that AQP4-IgG^+^ NMO is refractory to glucocorticoid treatment, and produces extensive visual field loss from the optic nerve and optic chiasma to the optic tract (2).

AHRU is a rare disease first reported by Delancy and Hitch (3). From 1994 to 2019, 38 (4), 25 (5), 19 (6), and 6 (7) cases in Japan, South Korea, Taiwan, and Thailand, respectively, have been reported. These studies summarized the clinical features of AHRU with acute rectal ulcers, painless, massive rectal bleeding, and negative blood and stool culture. The pathological features of AHRU present as necrosis with denudation of the covering epithelium, hemorrhage, and multiple thrombi in the vessels of the epithelium and underlying stroma (5, 6). Hospitalization, antithrombotic drug use, and lower serum albumin value are risk factors for AHRU (4); however, the pathogenesis of AHRU remains largely unknown.

CMV has been well-recognized as a pathogen causing opportunistic infections in immunocompromised patients such as those infected by human immunodeficiency virus (HIV) as well as recipients of solid organ or hematopoietic stem cell transplant (8–10). The prevalence of CMV seropositivity in human populations ranges from 50 to 95% (11). Primary infection with CMV in healthy individuals is usually clinically silent and enters a life-long latency in most people (11). However, CMV reactivation occurs in approximately one-third of critically ill immune-competent patients and is associated with worse outcomes, including prolonged hospitalization and death. In critically ill immune-competent patients, most CMV reactivation occurs between 4 and 12 days after admission to an intensive care unit (ICU) (8). Rare instances of enteritis (12) and NMO (13) caused by CMV in immune-competent individuals have been reported.

In this work, we report a co-NMO and AHRU case associated with CMV infection and review the literature on published CMV-NMO and CMV-AHRU patients.

## Case Presentation

**Figure 1:**
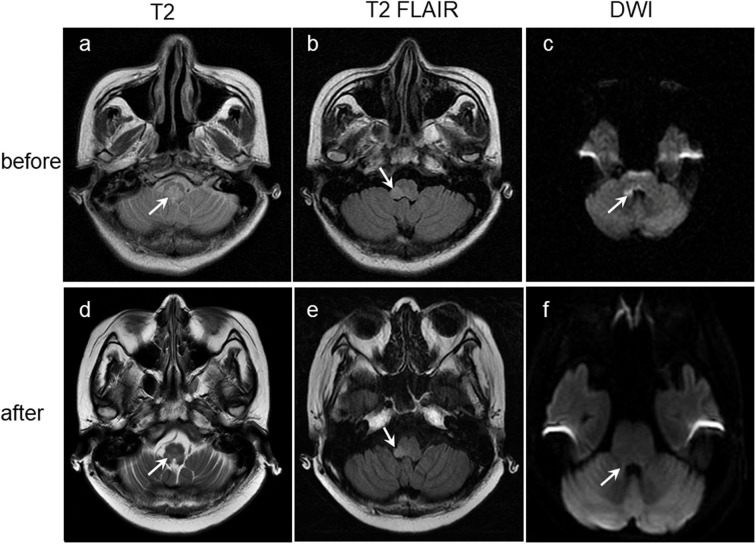
Lesions (arrowed) on brain coronary MRI images before **(a–c)** and after **(d–f)** antiviral treatment showing obviously manifested shrinkage after antiviral treatment. T2-weighted: Lesions on the medulla oblongata **(a,d)**. T2-FLAIR-weighted: Lesions on the right arm of medulla oblongata **(b,e)**. DWI: Lesions on right side of the right corpora quadrigemina, right side wall of the fourth ventricle and right side of the medulla oblongata **(c,f)**. DWI, Diffusion-weighted MRI.

A 40-year-old ethnic Han Chinese female, who used to be healthy and had no history of disorders related to the nervous system, respiratory system, circulatory system, digestive system, rheumatic immune system, and also had no history of mental disorders, from a family without history of genetic disorders, hypertension, coronary heart disease, diabetes, genetic inherited diseases, etc., forming a happy family with her beloved husband and two healthy daughters, initially presented with intractable nausea and dizziness for 9 weeks. She went to local hospital. Full blood count, serum biochemical tests, gastroscopy and head CT examination showed no obvious abnormalities. She felt that her nausea and dizziness did not improve after symptomatic therapy and even followed by dysphagia, limb weakness, blurred vision, ghosting in the right eye, and fatigue. Two weeks later, she went to another local hospital. A blood test was positive for AQP4 antibodies and brain MRI found high-signal lesions (Figures 1a–c). She was diagnosed with NMO and treated with high dose of intravenous methylprednisolone. Two days later, she felt shortness of breath, hard to swallow and difficulty in walking. She was transferred to the ICU and received a tracheotomy and mechanical ventilation due to severe breathing difficulties, and a nasogastric tube feeding due to difficulty swallowing. Her condition did not improve over 2 weeks despite treatment with high-dose intravenous methylprednisolone (0.5 g per day for 3 days, 0.25 g per day for 3 days, 0.12 g per day for 2 days) and immunoglobulin (20 g/day for 5 days) followed by 3 rounds of plasma exchange.

She was transferred to our hospital at midnight, and physical examination revealed a temperature and blood pressure of 37.1°C and 96/68 mmHg, respectively. She was conscious on mechanical ventilation, with blurred vision of the right eye, shallow right nasolabial fold, upper limb muscle strength level 1, and lower limb muscle strength level 0. Laboratory tests revealed a white blood cell (WBC) count of 28,240 cells/μL, serum albumin 32.3 g/L, prothrombin time 16 s, activate partial thrombin 38.6 s, and D-dimer 2.24 μg/mL. Rheumatoid, lupus, and vasculitis-related immune indicators, and biochemical indicators related to the heart, liver, and kidney function showed no obvious abnormalities. Tuberculosis-specific enzyme-linked immunospot assay (T-SPOT), hepatitis B virus, hepatitis C virus, HIV, CMV-DNA, CMV-IgM, and Epstein-Barr virus (EBV)-IgM were negative; however, the CMV-IgG antibody was positive. Alpha-fetoprotein, carcinoembryonic antigen, and carbohydrate antigen (CA) 19-9, 125, 153, 50, 724, and 242 were negative. Blood and CSF smears and cultures were negative.

**Figure 2:**
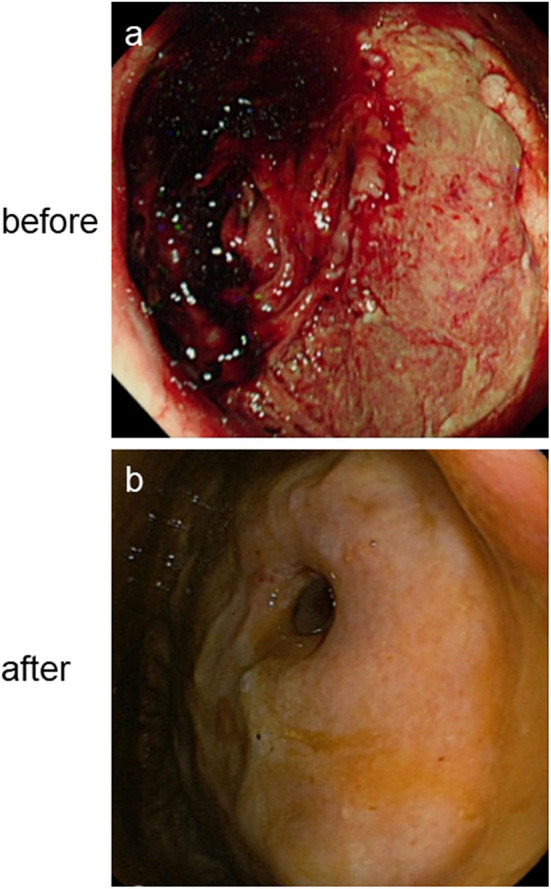
Changes of rectal ulcer on colonoscopy before **(a)** and after **(b)** antiviral treatment.

On day 2 of admission, the patient developed 150 ml suspicious menstrual blood, accompanied by a high fever, and marked increase in C-reactive protein (CPR) and procalcitonin (PCT); however, WBC count, hemoglobin concentration (1, 3), -β-D-glucan and galactomannan (GM) tests, and liver and kidney function were not significantly altered. Chest CT showed new glass-like lesions on both upper lungs. Multiple blood, sputum, and urine specimens were taken for pathogenic cultures (all final culture results were negative). Considering the hospital stay and administration of high doses of hormones in the external hospital, the patient was diagnosed with pulmonary infection and underwent an empirical broad-spectrum antibacterial and fungal treatment with meropenem, linezolid, and caspofungin. On day 3 of admission, the patient's body temperature peak began to decline; however, she suddenly passed 640 ml dark red bloody stool painlessly, resulting in shock. Blood transfusion and drug hemostasis treatment were administered to treat the shock. Meanwhile, bedside gastroscopy, colonoscopy, and full abdominal CTA scan were performed to identify the cause of bleeding. Abdominal CTA found a high-density shadow in the colon and rectum, which was considered an accumulation of blood; however, no bleeding vessels were found. Gastroscopy found no lesions in the upper digestive tract. However, colonoscopy revealed a large ulcer-like lesion at 10 cm above the anus and a large amount of feces and dark red bloody fluid (Figure 2a). Biopsy of ulcer-like lesions could not be performed due to coagulation dysfunction. On day 4 post-admission, the patient's body temperature returned to normal. On day 7 post-admission, the WBC count, CRP, PCT, and chest CT were normal; therefore, the anti-infective agents were removed; however, the daily excretion of 200–800 ml dark red bloody stool of this patient continued, and her muscle strength and vision loss did not improve.

Based on the patient's bleeding characteristics and lesions found via colonoscopy, it was speculated that the cause of the patient's acute lower gastrointestinal bleeding was most likely AHRU. The pathogenesis of AHRU remains largely unknown. Therefore, we speculated that the presence of AHRU in this patient may be linked with her NMO.

To assess this link, we reviewed literatures and only found one reported case of an acute NMO with rhabdomyolysis in an immune-competent patient following CMV infection (13). Infectious pathogens may trigger and exacerbate NMO by prompting AQP4-IgG production (14). AQP4-IgG+ NMO is refractory to glucocorticoid treatment. In this case, the patient did not respond to high-dose intravenous methylprednisolone and immunoglobulin followed by plasma exchange. Therefore, we speculated that NMO in this case was caused by the CMV infection.

**Figure 3:**
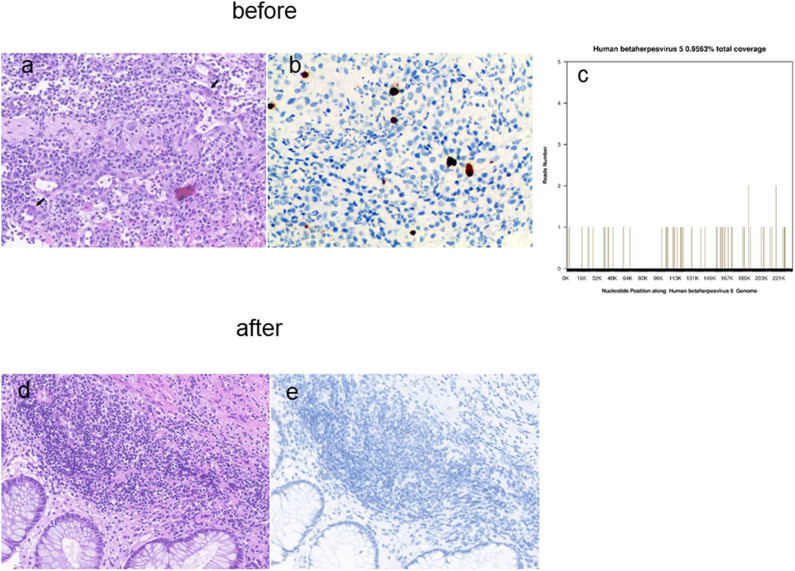
CMV HE staining and IHC of the biopsy specimen from the edge of the rectal ulcer lesions before **(a,b)** and after **(d,e)** antiviral therapy. Detection of CMV gene copies in CSF by NGS. Magnification × 400 **(c)**. HE, Hematoxylin and eosin; IHC, immunohistochemical; NGS, next-generation sequencing.

Thereafter, we wondered whether CMV infection could induce AHRU. Enteritis (12) caused by CMV in immune-competent individuals had been reported, although very rarely. Interestingly, no relevant literature about CMV-AHRU was found. However, ARHU may be caused by CMV infection from a monistic point of view. To verify this, on day 23 post-admission, CMV inclusions of pathology of a mucosal tissue specimen from the edge of the patient's rectal ulcer was carried out using routine HE staining and IHC. These results confirmed the presence of CMV infection (Figures 3a,b). In addition, CSF pathogen gene sequencing also detected CMV gene copies on day 25 post-admission (Figure 3c) and serum AQP4-IgG was still positive.

After 2 weeks of treatment with ganciclovir and sodium phosphinate, the patient's nausea, dizziness, visual impairment, and limb weakness gradually improved. After 3 weeks of antiviral therapy, her lower gastrointestinal bleeding stopped, and the CMV staining of rectal biopsies (Figures 3d,e), CSF CMV gene sequencing, and serum AQP4 all became negative. Further, high-signals on the brain MRI were significantly reduced (Figures 1d–f). After 4 weeks of antiviral therapy, the colonoscopy revealed a smooth intestinal wall at the original lesion (Figure 2b). In addition, her right eye visual acuity was resumed to counting fingers at 2 m, the muscle strength of her upper and lower limbs recovered to level 4 and 3, respectively. Following this, the antiviral drugs were stopped and the patient was discharged back to her local hospital for further rehabilitation.

## Discussion

Lesions on brain MRI and positive AQP4-IgG in this patient fulfilled the 2015 diagnostic criteria of NMO (15). Neuromyelitis optica spectrum disorder (NMOSD) is a highly specific serum autoantibody against AQP4 (1). AQP4 is an osmosis-driven, bidirectional water channel found on surface of astrocyte. AQP4-IgG presents in 65–88% of patients with NMOSD. In AQP4-IgG seropositive NMO, AQP4-IgG binds to AQP4 resulting in complement-dependent astrocyte injury and secondary inflammation, demyelination, and neuron loss (16). The sites of high AQP4 expression correlates with the preferred lesion sites in NMO (17). Zhong et al. reviewed that exposure to the organisms *Mycobacterium tuberculosis, Helicobacter pylori, Chlamydophila pneumonia, Clostridium perfringens*, and *herpes simplex virus-1* might be associated with AQP4-IgG^+^ NMO (18). The positive result of CMV in the pathological biopsy and CSF showed a correlation with the serum AQP4-IgG positive in this patient. However, the precise mechanisms behind the release of anti-AQP4 antibodies following a CMV activation or reactivation remains unknown. Infectious pathogens may potentially trigger or exacerbate NMO by several potential mechanisms, such as by prompting AQP4-IgG production (18). Pathogens and related antigens influence on the Th17 and regulatory T-cell (Tregs) balance and prompt a systemic inflammatory response by activating and recruiting neutrophils and monocytes, which increasing the level of cytokines and leading to the disruption of the blood-brain barrier (BBB), letting more T cells and AQP4-specific antibodies into the CNS (19–21). AQP4-IgGs enter the CNS and increase BBB permeability and bind to aquaporin-4 channels. Thereafter, AQP4-IgG-AQP4 activates complements, chemotactic signals, and immune cell infiltration, and alters CNS water homeostasis (22). Approximately two thirds of NMOSD patients are associated with AQP4-IgG and complement-mediated damage to the central nervous system (1).

The standard treatment for acute NMO is high-dose intravenous corticosteroids. For unresponsive patients, plasmapheresis is the treatment of choice (22); however, AQP4-IgG^+^ NMO is refractory to glucocorticoid treatment (13). Our patient was not responsive to steroids or plasma exchange. However, her fatigue, visual impairment, and lesions on brain MRI were all improved after anti-CMV treatment, and the serum AQP4 antibody also turned negative. Therefore, we speculated that her AQP4-IgG+ NMO may be related with CMV infection. Meanwhile, we wondered whether AHRU in this patient was related to CMV infection. Surprisingly, literature review did not reveal any positive studies. This is the first report of simultaneous occurrence of NMO and AHRU in a CMV infected patient. CMV infection can be asymptomatic in immuno-competent individuals. Careful histopathologic review is critical in the efficient detection of CMV by a pathologist. CMV inclusions can be identified on a routine HE staining, and IHC is available to identify CMV in the tissue (23). Interestingly, when cells are stained positive, serum CMV viral loads could be positive or negative (24). This patient's serum viral load was negative; however, CMV was detected on both, the routine HE staining (Figure 3a) and IHC (Figure 3b) of a mucosal tissue specimen from the edge of the patient's rectal ulcer.

The current gold standard for genotypic detection of CMV drug resistance is Sanger sequencing of PCR-amplified UL97 and UL54 gene segments. However, this approach often fails for viral loads of <1,000 CMV copies/ml of patient plasma (25). Recent studies have successfully used next-generation sequencing (NGS) platforms to study CMV intrahost population diversity in clinical specimens and CSF diagnostics in infectious diseases (26). The CSF smear and culture were negative in this case; however, CMV gene copies were detected by CSF pathogen gene sequencing (Figure 3c).

Although the specific pathogenic mechanism of CMV infection in NMO and AHRU is yet to be studied, the benefits of antiviral therapy in CMV-NMO and CMV-AHRU are also unclear; however, the results of HE and IHC staining of the biopsy specimen from the edge of the ulcer lesion and CSF NGS suggested that CMV infection may be associated with AHRU and NMO in this patient. And the improvement of the patient's clinical manifestations, laboratory examinations results, auxiliary examinations results and histopathological examination results related to NMO and AHRU including gastrointestinal bleeding, nausea, dizziness, visual impairment, and limb weakness, disappearance of AQP4 antibodies in the blood and CMV genes in the CSF, the healing of rectal ulcer on colonoscopy and shrinkage of lesions on brain MRI, clearance of CMV in rectal pathological biopsy tissues, indicating a causal role of CMV infection in NMO and AHRU.

## Ethics Statement

Written informed consent was obtained from the patient and his spouse for the participation in the study and the publication of this report in accordance with the Declaration of Helsinki. The case report is exempt from institutional review board approval.

## Author Contributions

JL is mainly responsible for the diagnosis and treatment of the case and the writing of this paper. XS is responsible for collating the relevant data for the article. YL is responsible for the colonoscopy. NC is responsible for the pathological diagnosis. YS is responsible for accompanying the patient's husband to the local hospital to apply for the patient's brain MRI images and image editing. YW is the deputy chief physician in the patient's treatment team. B-QW is the chief physician of the patient diagnosis and treatment team, and the PI of the research team. All authors read and approved the final manuscript.

## Conflict of Interest

The authors declare that the research was conducted in the absence of any commercial or financial relationships that could be construed as a potential conflict of interest.

## References

[1] Pittock SJ Berthele A Fujihara K Kim HJ Levy M Palace J. Eculizumab in aquaporin-4-positive neuromyelitis optica spectrum disorder. N Engl J Med. (2019) 381:614–25. 10.1056/NEJMoa1900866 31050279

[2] Kezuka T. [Optic neuritis–immunological approach to elucidate pathogenesis and develop innovative therapy]. Nippon Ganka Gakkai Zasshi. (2013) 117:270–91. 23631257

[3] Delancy H Hitch WS. Solitary rectal ulcer a cause of life-threatening hemorrhage. Surgery. (1974) 76:830–2. 4547619

[4] Komai T Omata F Shiratori Y Kobayashi D Arioka H. Risk factors for acute hemorrhagic rectal ulcer syndrome and its prognosis: a density case-control study. Gastroenterol Res Pract. (2018) 2018:8179890. 10.1155/2018/8179890 30158969 PMC6109505

[5] Jung JH Kim JW Lee HW Park MY Paik WH Bae WK. Acute hemorrhagic rectal ulcer syndrome: comparison with non-hemorrhagic rectal ulcer lower gastrointestinal bleeding. J Digest Dis. (2017) 18:521–8. 10.1111/1751-2980.12513 28753222

[6] Tseng CA Chen LT Tsai KB Su YC Wu DC Jan CM. Acute hemorrhagic rectal ulcer syndrome: a new clinical entity? Report of 19 cases and review of the literature. Dis Colon Rectum. (2004) 47:895–903. 10.1007/s10350-004-0531-1 15129312 PMC7177015

[7] Maneerattanaporn M Pongpaibul A Pongprasobchai S Kachintorn U Manatsathit S. Acute hemorrhagic rectal ulcer syndrome: the first case series from Thailand. J Med Assoc Thai. (2012) 95(Suppl. 2):S48–55. 22574529

[8] Limaye AP Kirby KA Rubenfeld GD Leisenring WM Bulger EM Neff MJ. Cytomegalovirus reactivation in critically ill immunocompetent patients. JAMA. (2008) 300:413–22. 10.1001/jama.300.4.413 18647984 PMC2774501

[9] Smith DM Nakazawa M Freeman ML Anderson CM Oliveira MF Little SJ. Asymptomatic CMV replication during early human immunodeficiency virus (HIV) infection is associated with lower CD4/CD8 ratio during HIV treatment. Clin Infect Dis. (2016) 63:1517–24. 10.1093/cid/ciw612 27601222 PMC5106612

[10] Meesing A Razonable RR. Pharmacologic and immunologic management of cytomegalovirus infection after solid organ and hematopoietic stem cell transplantation. Expert Rev Clin Pharmacol. (2018) 11:773–88. 10.1080/17512433.2018.1501557 30009675

[11] Bate SL Dollard SC Cannon MJ. Cytomegalovirus seroprevalence in the United States: the national health and nutrition examination surveys, 1988-2004. Clin Infect Dis. (2010) 50:1439–47. 10.1086/652438 20426575 PMC11000537

[12] Karigane D Takaya S Seki Y Mastumoto Y Onose A Kosakai A. Cytomegalovirus enteritis in immunocompetent subjects: a case report and review of the literature. J Infect Chemother. (2014) 20:325–9. 10.1016/j.jiac.2013.12.004 24751234

[13] Tran C Du Pasquier RA Cavassini M Guex-Crosier Y Meuli R Ciuffreda D. Neuromyelitis optica following CMV primo-infection. J Intern Med. (2007) 261:500–3. 10.1111/j.1365-2796.2007.01794.x 17444889

[14] Muroishi T Sakai K Yanase D Ikeda Y Machiya T Kato-Motozaki Y. Serum anti-aquaporin-4 antibody-positive neuromyelitis optica spectrum disorder presenting as acute eosinophilic encephalomyelitis. J Clin Neurosci. (2018) 48:93–4. 10.1016/j.jocn.2017.10.074 29137920

[15] Wingerchuk DM Banwell B Bennett JL Cabre P Carroll W Chitnis T. International consensus diagnostic criteria for neuromyelitis optica spectrum disorders. Neurology. (2015) 85:177–89. 10.1212/WNL.0000000000001729 26092914 PMC4515040

[16] Duan T Smith AJ Verkman AS. Complement-dependent bystander injury to neurons in AQP4-IgG seropositive neuromyelitis optica. J Neuroinflamm. (2018) 15:294. 10.1186/s12974-018-1333-z 30348195 PMC6198534

[17] Wang F Liu Y Duan Y Li K Brain MRI abnormalities in neuromyelitis optica. Eur J Radiol. (2011) 80:445–9. 10.1016/j.ejrad.2010.06.024 20884147

[18] Zhong X Zhou Y Lu T Wang Z Fang L Peng L. Infections in neuromyelitis optica spectrum disorder. J Clin Neurosci. (2018) 47:14–9. 10.1016/j.jocn.2017.10.005 29066232

[19] Varrin-Doyer M Spencer CM Schulze-Topphoff U Nelson PA Stroud RM Cree BA. Aquaporin 4-specific T cells in neuromyelitis optica exhibit a Th17 bias and recognize *Clostridium* ABC transporter. Ann Neurol. (2012) 72:53–64. 10.1002/ana.23651 22807325 PMC3405197

[20] Banati M Csecsei P Koszegi E Nielsen HH Suto G Bors L. Antibody response against gastrointestinal antigens in demyelinating diseases of the central nervous system. Eur J Neurol. (2013) 20:1492–5. 10.1111/ene.12072 23293933

[21] McColl BW Rothwell NJ Allan SM. Systemic inflammation alters the kinetics of cerebrovascular tight junction disruption after experimental stroke in mice. J Neurosci. (2008) 28:9451–62. 10.1523/JNEUROSCI.2674-08.2008 18799677 PMC6671112

[22] Bruscolini A La Cava M Mallone F Marcelli M Ralli M Sagnelli P. Controversies in the management of neuromyelitis optica spectrum disorder. Expert Rev Neurother. (2019) 19:1127–33. 10.1080/14737175.2019.1648210 31339052

[23] Juric-Sekhar G Upton MP Swanson PE Westerhoff M. Cytomegalovirus (CMV) in gastrointestinal mucosal biopsies: should a pathologist perform CMV immunohistochemistry if the clinician requests it? Hum Pathol. (2017) 60:11–5. 10.1016/j.humpath.2016.09.009 27666768

[24] Liao X Reed SL Lin GY. Immunostaining detection of cytomegalovirus in gastrointestinal biopsies: clinicopathological correlation at a large academic health system. Gastroenterol Res. (2016) 9:92–8. 10.14740/gr725e 28058077 PMC5191896

[25] Sahoo MK Lefterova MI Yamamoto F Waggoner JJ Chou S Holmes SP. Detection of cytomegalovirus drug resistance mutations by next-generation sequencing. J Clin Microbiol. (2013) 51:3700–10. 10.1128/JCM.01605-13 23985916 PMC3889754

[26] Simner PJ Miller HB Breitwieser FP Pinilla Monsalve G Pardo CA Salzberg SL. Development and optimization of metagenomic next-generation sequencing methods for cerebrospinal fluid diagnostics. J Clin Microbiol. (2018) 56:e00472–18. 10.1128/JCM.00472-18 29976594 PMC6113476

